# ISFET pH Sensitivity: Counter-Ions Play a Key Role

**DOI:** 10.1038/srep41305

**Published:** 2017-02-02

**Authors:** Kokab B. Parizi, Xiaoqing Xu, Ashish Pal, Xiaolin Hu, H. S. Philip Wong

**Affiliations:** 1 Department of Electrical Engineering, Stanford University, Stanford, CA 94305, USA

## Abstract

The Field Effect sensors are broadly used for detecting various target analytes in chemical and biological solutions. We report the conditions under which the pH sensitivity of an Ion Sensitive Field Effect transistor (ISFET) sensor can be significantly enhanced. Our theory and simulations show that by using pH buffer solutions containing counter-ions that are beyond a specific size, the sensor shows significantly higher sensitivity which can exceed the Nernst limit. We validate the theory by measuring the pH response of an extended gate ISFET pH sensor. The consistency and reproducibility of the measurement results have been recorded in hysteresis free and stable operations. Different conditions have been tested to confirm the accuracy and validity of our experiment results such as using different solutions, various oxide dielectrics as the sensing layer and off-the-shelf versus IC fabricated transistors as the basis of the ISFET sensor.

The ISFET has been used for many years to measure the pH value of the electrolyte solutions^1^,^2^,^3^,^4^,^5^,^6^,^7^. It was first introduced by Bergveld in the 1970 s^1^. In the original structure of an ISFET, the gate oxide is in direct contact with the electrolyte solution, therefore, acting as a sensing dielectric. Extended Gate ISFET (EG-ISFET) is a modified version of the ISFET in which, the sensing oxide is decoupled from the gate oxide by using an extended conductive layer^8^,^9^,^10^,^11^,^12^,^13^. Since the gate oxide is protected from the electrolyte solution, it creates a more robust structure for the long time measurements inside the liquid_8,9_. Our experimental sensor is an EG-ISFET; the sensing gate is formed by extending out the gate of an nFET transistor and depositing a thin Al_2_O_3_ layer on the extended gate as the sensing dielectric. In this structure, the area of Sensing Gate (SG) compared to the area of entire sensor is one of the structural design parameters that greatly influences the electrical behavior of the sensor due to the existence of parasitic capacitance which degrades the measured sensitivity of the sensor (in Supplementary information section I, we elaborate more on this). The buildup of surface charge due to the protonation/deprotonation reactions induces a potential at the sensor surface. Through the effective coupling capacitance between the sensor surface and Floating Gate (FG), the surface potential modulates the potential of the FG, and therefore there will be a corresponding shift in threshold voltage (V_T_) of the sensor^12^,^13^. According to the Boltzmann distribution model for the H^+^ ions^14^,^15^,^16^, the pH value at the sensor surface is^14^:


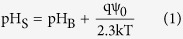


where the subscript S and B denote the pH at the sensor surface and in the bulk solution, Ψ_0_ is the potential drop across the diffusion layer. In eq. 2, the intrinsic buffer capacity (*β*_*int*_) is defined as the ability of collecting charge at the sensor surface (*σ*_*0*_) due to the change in surface pH (*pH*_*S*_)^14^:


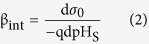


And the diffusion capacity (*C*_*diff*_) is the ability of storing the opposing charge in solution near the surface due to the change in surface potential^14^:


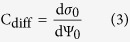


Therefore, we can write:


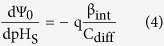


Differentiating eq. 1 with respect to the bulk pH and using eq. 4, the sensitivity of surface potential to the bulk pH can be derived as^14^:


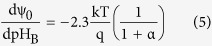


with


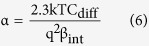


here, α is a dimensionless sensitivity parameter with a positive value. Therefore, the sensitivity of potential at the sensor surface and the corresponding change of the sensor threshold voltage to the bulk pH are limited to 2.3 kT/q = 59 mV/pH (Nernst limit). There have been many attempts to enhance the sensitivity of the ISFET sensors^17^,^18^,^19^,^20^,^21^,^22^,^23^,^24^. Engineering the sensing surface^17^,^18^, reducing the screening of counter-ion charge^19^, electromechanical coupling techniques^20^, engineering the structural dimensions^21^ and amplification of signal through the dual-gated structures^22^,^23^,^24^ are among those that have been reported so far. As we discussed, the sensitivity limitation originates from the classic Boltzmann distribution of hydrogen ions for deriving the pH sensitivity^14^,^15^,^16^. However in reality, this model predicts an extremely large number of counter-ions near the surface when they are subjected to a large voltage^25^,^26^. The concentration of counter-ions can even exceed the maximum number of ions that can actually fit onto the surface. The reason is that the Boltzmann model assumes ions are point-like charges with zero physical sizes (Fig. 1a)^27^,^28^. This phenomena that the ion concentration gets saturated at the surface beyond a critical potential (Ψ_C_) is known as crowding or steric effect which was reported in^25^,^26^. The term Ψ_C_ has been used as a representative for both critical and steric potential throughout this text. The crowding effect strongly changes the potential profile of the electrolyte diffusion layer. For the potential values larger than Ψ_C_, the counter-ions repel themselves and form a wider diffusion layer which results in a lower ion concentration at the surface than predicted by the point-charge assumption (Fig. 1b)^26^. The capacitance across the diffusion layer reduces in response to the applied voltage which is in contrast with the classic Boltzmann model as it predicts they will exponentially rise with the applied bias (Fig. 1c)^26^,^29^.

A modified Boltzmann model has been proposed which takes into account the effect of ion sizes^29^. The surface concentration (*c*_*S*_) of the counter-ions with the bulk concentration of c_B_ as a function of the electrostatic potential (Ψ_0_) and the ion size (*a*) is given by the modified Boltzmann distribution^29^:


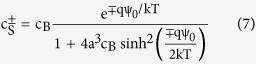


On the other hand, for a buffer solution, due to its pH restoration property^30^, the crowding effect of the counter-ions results in higher hydrogen ion concentration at the sensor surface and therefore larger pH sensitivity, as explained below. The buffer solutions usually are made of similar quantities of weak acids and their conjugate base salts to counteract small changes in pH^30^. The chemical reactions in a pH buffer solution can be mainly summarized in these below equations:


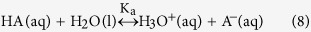



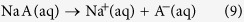



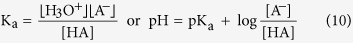


where HA is the weak acid and NaA is its conjugate base salt. K_a_ is the acid dissociation constant which determines how strongly an acid is ionized in water^31^. K_a_ is much smaller than 1 for weak acids which means there would be large amount of weak acid left together with the conjugate ions in the solution for the pH restoration.

By substituting the size dependent ion concentrations from the modified Boltzmann model into the logarithmic version of eq. 10, the surface pH value can be obtained as below:


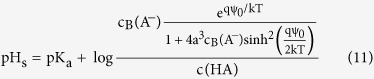


Where *c*_*B*_(*A*^*−*^) is concentration of counter-ion in the bulk solution and *c(HA*) is the concentration of acid molecules in solution. The pH value in the bulk solution can be written as:


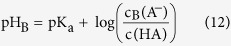


Therefore eq. 11 is simplified to:


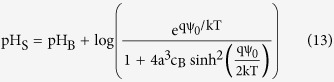


Similar to the Boltzmann model case, differentiating eq. 13 with respect to the bulk pH and using eq. 4, the sensitivity of surface potential to the bulk pH can be derived as below (the step by step derivations are given in section II of the Supplementary information):


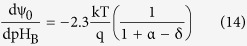


With


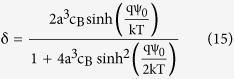


where δ is a dimensionless sensitivity parameter between zero and one. By comparing the sensitivity in eq. 14 with the sensitivity from the classic Boltzmann model (eq. 5), we can see the sensitivity of the potential at the sensor surface not only depends on the potential drop across the diffusion layer, but it also depends on the sizes of the buffer ions. As the counter-ion size increases, the δ value goes higher. A large enough counter-ion size will boost the δ value high enough to push the pH sensitivity above 59 mV/pH. To see clearly how the sensitivity parameters of α and δ changes the sensitivity, their simulated values and obtained sensitivity using these parameters for various ion sizes are given in section III of the Supplementary information. Our model loses its validity when there is large amount of counter-ions collected at the sensor surface compared to its initial value in the bulk solution^25^,^29^. However, this would happen at relatively large potentials due to the steric effect of counter-ions and their weak relation to the surface potential^25^,^29^. In the next part, we report a series of simulation and experiment results to confirm the effect of counter ion size on pH sensitivity. We first describe the structural model and simulation results, then we explain the sensor implementation and the electrical results.

## Results

### Models and simulation results

**Figure 2:**
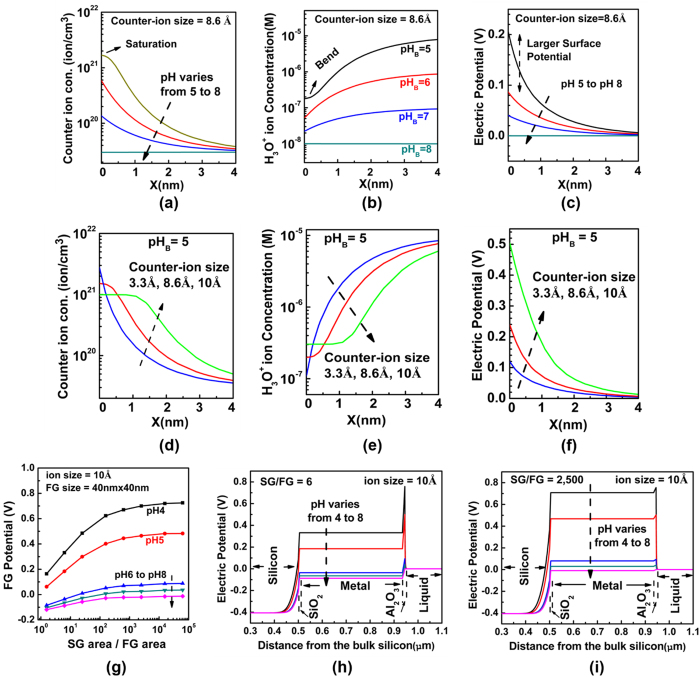
A simulated situation showing the effect of counter-ion crowding on the sensor sensitivity. At fixed counter-ion size = 8.6 Å, when pH_B_ = 5, the counter-ion concentration saturates near the sensing oxide surface (**a**), the surface H^+^ concentration bends and does not follow the Boltzmann model (**b**) and therefore larger electric potential occurs at sensor surface (**c**). At fixed pH_B_ = 5, when the ion size is larger, there is larger decrease on counter-ion surface concentration (**d**) and therefore greater increase on hydrogen ion surface concentration (**e**) which leads to higher electric potential at surface (**f**). The FG potentials are shown for various SG/FG area ratios at constant counter-ion size = 10 Å (**g**), there is larger coupling between the sensor surface potential and FG when the SG/FG ratio is larger. The electrostatic potential across the sensor when SG/FG = 6 (**h**) and SG/FG = 2,500 (**i**) are compared.

We used COMSOL Multi-Physics simulator^32^ for the modeling of our sensor system. The simulation structure consists of an EG-ISFET sensor in contact with the electrolyte solution. The bulk silicon doping is set to 1 × 10^16^ cm^−2^, the gate oxide is 10 nm thick SiO_2_ and the gate electrode is assumed to be a pure metal extended outside to form the sensing membrane. The 20 nm Al_2_O_3_ layer is placed on top of the extended gate electrode as the sensing dielectric while the pH of point of zero charge (pH_pzc_) is set to pH 8. The pH solution is assumed to be a buffer with the bulk concentration of *c*_*0*_ = 50 mM. The sensitivity of FG potential to the change in bulk pH solution is considered as the pH sensitivity of the sensor and all the ion sizes are considered to be their hydrated values in the solution. Figure 2 shows a scenario which demonstrates how the counter-ion crowding affects the sensor sensitivity. When the acid conjugate ion size is *a* = 8.6 Å, the maximum concentration at the sensor surface would be a^−3^ = 1.57 × 10^21^ cm^−3^. As shown in Fig. 2a, at pH 5 which is farther from pH_pzc_, the counter-ion concentration reaches to this maximum concentration at the surface while for the other pH values, the concentrations still increase exponentially. The early saturation at pH 5 is due to the higher surface charges which attract more counter-ions near to the surface. Because of the pH restoration property of the buffer solutions (eq. 8), saturation of the counter-ions increases the surface concentration of *H*^+^ ions as shown in Fig. 2b. As a result, a higher electric potential (Fig. 2c) builds up at the sensor surface. The results in Fig. 2d–f demonstrate how strongly the sensitivity of surface potential at pH_B_ = 5 is dependent on the ion size; when the ions are small (=3.3 Å), there is no steric effect of counter-ions at the sensor surface and therefore the sensitivity is low. But when the ions are large (=10 Å), the steric effect of counter-ions at the sensor surface strongly amplifies the pH sensitivity of surface potential. The sensitivity of sensor is maximized when the SG/FG area ratio is large (Fig. 2g). In this paper, we use the term SG/FG area ratio as a proxy of the parasitic capacitance, where a large SG/FG area ratio represents the case where parasitic capacitance effect is minimal. For the smaller ratios, the weak coupling between the sensor surface and FG cannot effectively transfer the surface potential to the FG potential (Fig. 2h) but as the SG/FG area ratio gets larger, the stronger coupling results in lower potential drop in the sensing oxide and therefore larger potential is seen at the FG (Fig. 2i).

The effect of different salt configurations in solution and also bulk pH value beside the buffer counter-ion size are also investigated in our simulation. The results are given in section IV and V of the Supplementary information respectively.

### Measurement and Electrical Results

**Figure 3:**
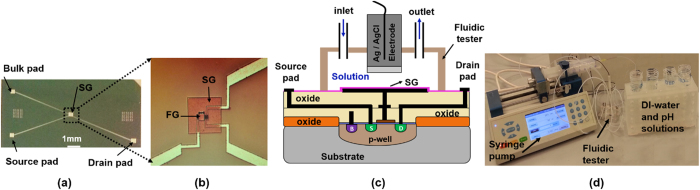
(**a**) Die photo of the fabricated sensor. (**b**) Zoom in view of the SG area. (**c**) The schematic of the fluidic tester mounted on top of the sensor and (**d**) the fluidic setup.

Our sensor structure is fabricated using the standard IC fabrication techniques. The fabrication steps start with making an n-type MOSFET using the conventional CMOS fabrication methods. The transistor source, drain and bulk electrodes are extended farther from the transistor channel for ease of access to the SG during the measurement using pH solutions. The SG electrode is formed by extending the gate of the n-FET transistor outside using Al metal followed by deposition of 20 nm ALD Al_2_O_3_ layer as the sensing dielectric. In order to measure the response of our sensor structure to different pH solutions, we use a fluidic test setup which enables us to pass the liquid through the sensing gate of the sensor during the measurements in real time. Figure 3 illustrates a die photo of the fabricated sensor together with the fluidic test setup.

**Figure 4:**
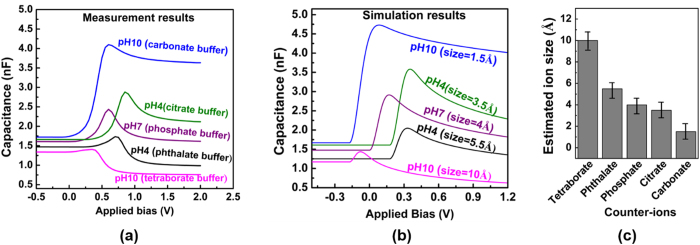
(**a**) The measurement result of capacitance-voltage behavior of an Ag electrode in contact with different buffer solution, (**b**) the predicted simulation result and (**c**) the estimated effective hydrated sizes for the counter-ions are used in this experiment.

The sensor response has been measured with different pH buffer solutions. Each of the buffer solution has a specific size for the counter-ions. Determining the effective hydrated size of the counter-ions is one of the challenges as the reported solid state ionic size cannot be applied in the liquid state. The effective hydrated size of the counter-ions can be significantly larger than its ionic diameter due to the formation of solvation shell and the larger ion to ion correlation in liquid^33^. Most studies of molecular dimensions have relied on measurements of the effective radius by scattering studies using neutrons or X-rays^34^,^35^. In this work, we use the measured capacitance-voltage behavior of an Ag electrode in contact with the electrolyte solutions to corroborate the simulation results and infer the counter-ion sizes. The measured capacitance results are shown in Fig. 4a. The capacitances first rise, reaching to a maximum at different biases and then fall down as voltage bias further increases. This behavior agrees with the prediction of the modified Boltzmann theory for the electrolyte diffusion capacitance near a biased surface (Fig. 4b). Comparing the simulation results with the measurement data, we estimate the range of the sizes for the counter-ions in our solutions (Fig. 4c). Using this method, the estimated size of the phosphate ion is about 4 Å which agrees well with the reported value^35^.

**Figure 5:**
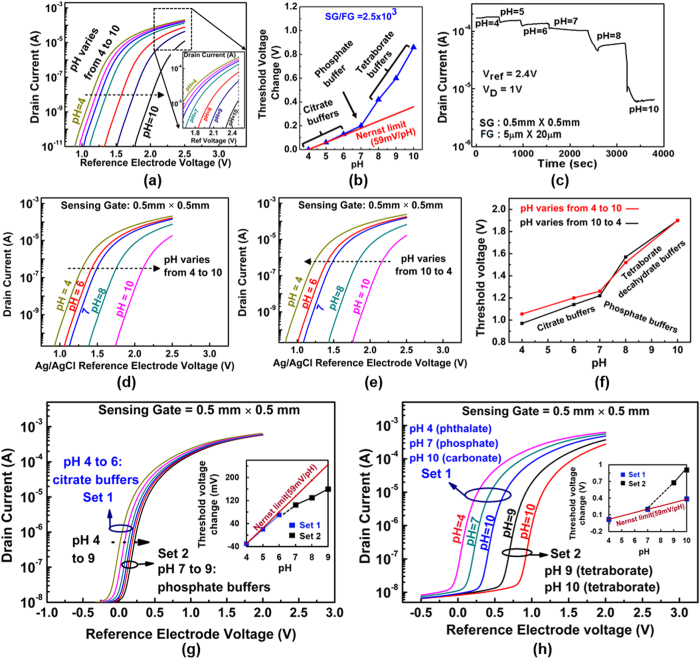
Measurement results of the sensor at fixed SG/FG area = 2500, (SG area = 0.5 mm × 0.5 mm, FG area = 5* *μm × 20 μm. 20 nm-thick ALD Al2O3 is used as the sensing dielectric). (**a**) The drain current as a function of the reference electrode voltage for pH values from 4 to 10, (**b**) The sensitivity of threshold voltage as a function of pH solutions and (**c**) the time dependent measurement of drain current when the solution pH is varied at a fixed reference electrode voltage (2.4 V) and drain bias (1 V). The threshold voltage shift for different pH is consistent when pH values were changed from (**d**) higher pH values to lower (10 to 4) and (**e**) lower pH values to higher (4 to 10). (**f**) The change in threshold voltage as function of pH. Measurement results with different sets of counter ions shows that the pH sensitivity can be quite small, even lower than the Nernst limit (**g**), the pH sensitivity varies for two solutions with the same pH values but different counter-ion sizes (**h**).

We did several experiments to elucidate the effect of different sizes of the counter-ions on the measured pH sensitivity of our EG-ISFET structure. Figure 5 demonstrates a situation that the sensor responds quite non-linearly when pH is changed from 4 to 10 when pH 4 to 6 buffers are citrate-based, pH 7 buffer is phosphate-based and pH 8 to 10 buffers are tetraborate-based buffers. The sensitivity is relatively low from pH 4 to 6 (estimated size of ~4 Å) while the sensitivity significantly goes up from 8 to 10 (estimated size of ~10 Å). Figure 5c illustrates the transient response of the sensor drain current over the time when the pH solution was changed from pH 4 to pH 10. The results confirm the stability and consistency of our experiment. The transient response tracks well the value of the drain current when the reference electrode voltage is 2.4 V (zoom-in image in Fig. 5a). The repeatability of the results is also confirmed by performing measurement both by changing the pH values from high to low (10 to 4, Fig. 5d) and from low to high (4 to 10, Fig. 5e). These results clearly show that the measured non-linearity of the pH sensitivity is not due to hysteresis or covalent binding of electrolyte oxide interfacial molecules. Figure 5g and h show the sensor response when the pH sensitivity is measured with pH solutions using different buffer counter-ions. Figure 5g illustrates a situation when the sizes of the solution counter-ions are not large enough to result in enhanced pH sensitivity. The measured sensitivity is even lower than 59 mV/pH. Figure 5h demonstrates a situation that the solutions with different sizes of counter-ions (larger and smaller than steric size) but with the same pH can produce quite different measured pH sensitivity.

We have also tested our sensor with different dielectric materials as the sensing oxide: HfO_2_, Si_3_N_4_ and TiO_2_. The results are given in Supplementary information section VI. The measurement results show similar nonlinear behaviors when the sensor is measured with different pH buffer solutions. It confirms that the nonlinear sensor response are not due to the chemical interactions that are specific between the Al_2_O_3_ sensing surface and solution ions. In Supplementary information section VII, we have provided additional experiment data to support more our claims on hysteresis free, stability and repeatability of our measurement data.

**Figure 6:**
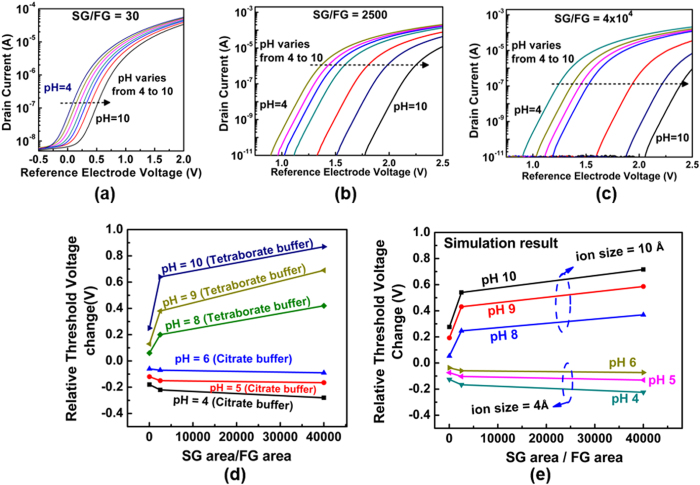
The experimental results of three different SG to FG size ratios. Sensitivity reaches its ideal value when the SG/FG size gets larger. (**a**), (**b**) and (**c**) show the drain current as a function of the reference electrode bias when the SG/FG ratios is 30, 2500 and 40,000 respectively. The extracted measured (**d**) and simulated (**e**) sensitivities versus the SG size.

We have also investigated the effect of the SG area on sensitivity of our structure. The electrical results of three structures with different SG to FG area ratios of 30, 2500 and 40,000 are shown in Fig. 6a,b and c respectively. Only the SG size has been changed here to vary the SG/FG ratio; the FG area is kept constant at 100 μm^2^. Regardless of the ion size, enlarging the SG/FG area ratio reduces the parasitic capacitance and therefore increases the measured pH sensitivity of the sensor structure (more about on the effect of SG/FG area ratio can be find in the Supplementary information section I). The sensitivity of the sensor vs the SG/FG ratio has been plotted in Fig. 6e. In this figure, pH 7 is used as the reference value to measure the change in the threshold voltage of the sensor. As it is seen, the SG/FG area dependence is more prominent for pH solutions with larger counter-ion sizes (pH 8 to 10, tetraborate buffers, size = ~10 Å) than those with smaller sizes (pH 4 to 6, citrate buffers, size = ~4 Å). This is due to the smaller electrolyte diffusion capacitance for the larger ions as the result of crowding effect (as given in Fig. 4a). These behaviors are similar to what the theory (Fig. 6e) predicts for the pH sensitivity of these buffer solutions. The simulation in Fig. 6e is carried out by including the salt ingredients of the buffer solutions used in this experiment (Supplementary information, section VIII) into our simulation model.

We have also made our extended gate ISFET structures using off-the-shelf transistors^21^ and the results were consistence with the results reported here.

## Discussion

The sensitivity limitation or Nernst limit originates from the classical Poisson Boltzmann model which assumes ions are point like charge with zero physical sizes. In this paper, we report the conditions under which the sensitivity of an extended gate ISFET pH sensor does not follow the Nernstian response and can be significantly enhanced. We emphasize the effect of analyte solution counter-ion size and electrolyte solution buffering capacity as the key roles in inducing the pH sensitivity at the sensor surface. Our model and simulation results indicate that for each buffer solution, beyond a specific size of the counter-ions, the crowding (steric) effect of the counter-ions at the sensor surface together with the buffering condition of the solution result in higher surface hydrogen ion activity. This leads to the larger pH sensitivity of the surface potential. The steric effect is more prominent for larger counter-ion sizes as those get closely packed at the surface at lower potentials. This is more noticeable for the pH values farther from pH_pzc_ due to higher surface charge which attracts more counter-ions near the surface. The existence of additional ions in solution especially when their ionic size are smaller than weak acid counter-ions, screens the buildup surface charge and therefore saturates the sensor sensitivity. The large SG/FG area ratio strengthens the capacitance coupling between the sensor surface and FG which can effectively transfer the larger sensitivity of the surface potential to the gate of the transistor. The proposed theory and model have been validated by a series of experiments. Repeatability and consistency of the experiment results are seen throughout the experiments. To test out the error free operations of our measurement system, the experiments have been carried out under different conditions such as use of various buffer solutions, implementing different oxide dielectrics as the sensing layer and use of off-the-shelf transistors as the basis of the ISFET sensor beside the IC-fabricated structures^20^. Understanding the dependency of pH sensitivity on the ion size helps us to better comprehend the performance of the sensors that are designed to work *in-vivo*/*in-vitro* applications.

## Materials and Methods

### COMSOL Simulation of EG-ISFET

The equations used in our simulation are listed in Tables 1 and 2. Table 1 includes the equations for each region of the structure: To model the interaction between the electrolyte solution and sensing oxide surface, the surface charge of the site binding model is assumed to be on top of the Al_2_O_3_. To account for the effect of the solution ion size, the modified Boltzmann model is used for representing the ion distributions in liquid. We assumed the substrate is a p-type bulk silicon, the gate oxide and sensing oxide are SiO_2_ and Al_2_O_3_ respectively without any trap charge inside, the electrode contacts work functions are zero and the reference electrode is a Faradaic electrode. The non-linear 2-D Poisson system is solved for calculating the potential and charge redistributions in our sensor system as a function of bulk pH and ion size. The structural mesh is highly refined at the sensor surface where the potential gradient is very steep, to ensure solution convergence. Table 2 includes the two additional conditions that were applied in our simulation model as the charge neutrality condition for the entire system and the solution buffering condition for the electrolyte region. The charge neutrality condition is used for balancing the charge between the electrolyte diffusion layer and silicon depletion area. The pH buffering condition in the electrolyte solution is obtained using the size dependent Hesselblach equation. It defines the pH value at each point of the solution as a function of ion size and ion concentration.

### Fabrication processes of EG-ISFET

The sensor structure is made on a bulk n-type silicon substrate with 5 to 10 Ω-cm resistivity. The following steps describe the fabrication process: The substrate surface doping is adjusted by a blanket ion implantation of 150 keV phosphorus ions with a dose of 1.5 × 10^13^ cm^−2^. The field oxide formation starts with growing 20 nm pad oxidation followed by a 200 nm Low Pressure Chemical Vapor Deposition (LPCVD) silicon nitride deposition. The nitride is then patterned and dry etched from all areas except the active regions. The 500 nm thick field oxide is wet thermally grown at 1000 °C for about 90 minutes. The nitride is then removed from the active area using the hot (150 °C) top phosphoric acid. The p-well implantation at the active region is done using the boron ions at 150 keV with a dose of 4.0 × 10^13^ cm^−2^. The 5 hours annealing at 1000 °C in N_2_ ambient is used to drive-in and activate the dopants. The 10 nm gate oxide is formed by dry thermal growth of SiO_2_ at 900 °C for about 20 minutes. Immediately after gate oxide growth, the 0.2 μm thick LPCVD polysilicon is deposited on the wafer. The dry etching process etches the patterned polysilicon defining the gate line of our structure. The self-aligned arsenic implantation at 50 keV with dose of 2 × 10^15^ cm^−2^ forms the doping for both polysilicon gates and n^+^ source/drain regions. The P^+^ bulk regions are doped using the BF_2_ implantation at 50 keV with dose of 2 × 10^15^ cm^−2^. The LPCVD LTO passivation layer of silicon oxide with thickness of 0.6 μm are deposited on wafer. The annealing step at 950 °C for 30 minutes is done for the purpose of both drive in the source/drain/bulk dopants after the implantation and also densification of Low Temperature Oxidation (LTO) passivation layer. The 0.6 um deep contact holes are then created into the oxide using the plasma etch providing access to the source/drain/bulk and polysilicon gate area. A 0.5 μm thick aluminum/silicon (1% silicon) alloy is deposited and patterned to form the source/drain/bulk and gate electrodes. Another layer of LPCVD LTO passivation with 2 μm thickness is deposited to isolate the first metal lines. The 2 μm deep contact holes are made through the second layer of passivation providing access to the gate electrode. The 1 μm thick aluminum metal is then deposited and patterned with different sizes to form the extended gate electrode. The fabrication ends with depositing the 20 nm Atomic Layer Deposition (ALD) Al_2_O_3_ layer as the sensing dielectric.

### Measurement setups

To test out our sensor structure under the liquid environment, a 7 mm wide fluidic tester was made using Computer Numeric Control (CNC) machining. Two 1 mm diameter holes placed on the lid for creating inlet and outlet to pass the liquid into the tester. The polyethylene tubes are then inserted into the inlet and outlet holes. The other end of the tubes are connected to a digital syringe pump and the solution media. The flow rate of 1 mL/S is used for our syringe pump during our experiment. The fluidic environment inside the tester is biased with a glass reference electrode with a frit junction on its tip. It consists of an Ag/AgCl internal electrode with the 3 M KCl filling solution. The reference tip outer diameter is 1.5 mm which is small enough to dip into our fluidic tester. The testing solution are all commercial pH buffered solutions purchased from Sigma Aldrich. The pH value for each solution is measured separately with a pH meter before use, to confirm the pH values. The electrical device characterizations (including drain current vs the reference bias for each pH solution and drain current vs. time while the pH varies) are all performed using the Agilent B1500A Semiconductor Device Analyzer. The capacitance measurements are done using the Agilent 1 MHz LCR meter. Before and after each measurement, the sensor is rinsed by pumping the DI water into the tester for at least 10 min. The electrical response is also measured 10 min after the desired solution is piped into the tester. The sensor test setup is kept unchanged under the measurement probes during each experiment.

## Additional Information

**How to cite this article**: Parizi, K. B. *et al*. ISFET pH Sensitivity: Counter-Ions Play a Key Role. *Sci. Rep.* **7**, 41305; doi: 10.1038/srep41305 (2017).

**Publisher's note:** Springer Nature remains neutral with regard to jurisdictional claims in published maps and institutional affiliations.

## Supplementary Material

Supplementary Information

## Figures and Tables

**Figure 1:**
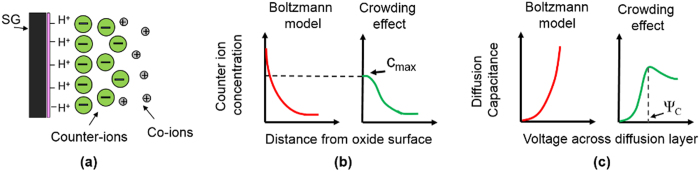
Crowding effect of the counter-ions near the charged sensing surface. (**a**) Schematic of distribution of non-zero size ions near the surface. (**b**) Limitation of the classic Boltzmann model due to the saturation of counter-ions at the surface, c_max_ is the maximum concentration of counter-ion near the surface. (**c**) The diffusion layer capacitance is reduced as a result of the crowding effect, Ψ_C_ is the steric potential. At Ψ_C_, the counter-ion concentration reaches to c_max_.

**Table 1:** The equations used for modeling the regions of the sensor system.

Si body	oxide	Sensor liquid interface	Liquid
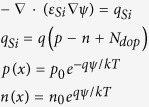 *n*_0_ = 10^10^ cm^−3^,*p*_0_ = 10^16^ cm^−3^*ε*_*Si*_ = 11.9 *ε*_*0*_	Gate oxide: 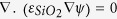 *ε*_SiO2_ = 3.9*ε*_0_Sensing oxide: 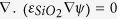 *ε*_*Al2*o3_ = 9*ε*_0_	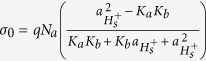 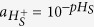 *N*_a_ = 8 × 10^14^ cm^−2^ (*K*_a_, *K*_b_) = (6, 10)	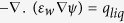 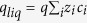 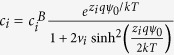 *v*_*i*_ = 2a^3^c_i_^B^,c_i_^B^ = 50 mM, *ε*_w_ = 80*ε*_0_

Where *ε*_*Si*_*, ε*_SiO2_, *ε*_w_, *ε*_*Al2*o3,_ *ε*_*w*_are the relative permittivity of silicon, gate oxide, sensing oxide and water respectively, *ε*_*0*_ is the vacuum permittivity. In column 1, *P(x*) and *n(x*) are the silicon hole and electron concentrations with the initial value of *p*_*0*_ and *n*_*0*_, *q*_*Si*_ is the total charge in bulk silicon, *N*_*dop*_ is the substrate doping. In column 3, *σ*_*0*_ is the sensing oxide surface charge density, *a*_*Hs*_ is the activity of hydrogen ion at the sensing oxide surface, *K*_*a*_ and *K*_*b*_ are in order the oxide association and dissociation constant and *N*_*a*_ is the oxide surface charge density. In column 4, *q*_*liq*_ is the solution total charge, *c*_*i*_ is the ion *i* concentration in solution with the bulk concentration of *c*_*i*_^*B*^. *z*_*i*_ is the ion *i* charge number, *a*_*i*_ is the ion *i* effective hydrated size and *v*_*i*_ is the ion *i* packing parameter.

**Table 2:** (a) Charge neutrality condition and (b) electrolyte pH buffering condition.

(a) Charge neutrality condition	(b) Buffer solution condition
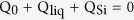 Surface charge: 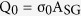 Liquid charge: 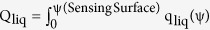 Silicon charge: 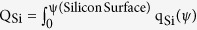	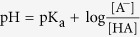 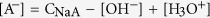 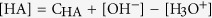 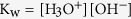

In column 1, *σ*_0_ is the sensing oxide surface charge density and A_SG_ is the SG area. In column 2, C_NaA_ is the initial conjugate salt concentration, C_HA_ is the initial acid concentration and K_w_ is the water dissociation constant.
