# Supplementary material for: ISFET pH Sensitivity: Counter-Ions Play a Key Role
Source: Sci Rep. 2017 Feb 2;7:41305. doi: 10.1038/srep41305 (PMC5288728; doi:10.1038/srep41305)
Supplement: Supplementary Information [file srep41305-s1.pdf]

ISFET pH Sensitivity: Counter-Ions Play a Key Role

Kokab B. Parizi[1], Xiaoqing Xu[1], Ashish Pal[1], Xiaolin Hu[1] and H. S. Philip Wong[1#]*

Supplementary information:

I. The capacitance model of EG-ISFET:

The schematic of an EG-ISFET and its capacitance model are given in Fig. S.1a and Fig. S.1b respectively.

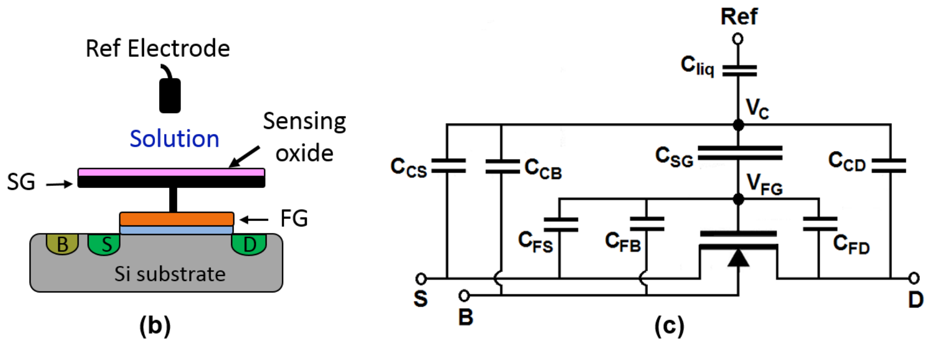

Supplementary Figure S.1. Schematic of (a) the EG-ISFET structure, SG is the Sensing Gate and FG is the Floating Gate. (c) The capacitance model of an EG-ISFET structure. V_C and V_{FG} are is the chemical and floating gate potentials respectively. C_{SG} is the SG dielectric capacitance. C_{FS}, C_{FD} and C_{FB} are in order the parasitic capacitances from the FG to the Source, Darin and Bulk electrodes. C_{CS}, C_{CD} and C_{CB} are the parasitic capacitances from the electrolyte-sensor surface to the Source, Drain and Bulk electrodes respectively. The FG and liquid areas are both constant in the simulation and the experiment.

A capacitance-based model that includes all the intrinsic and extrinsic capacitances (Fig. S.1b) is used to derive the FG potential. By using the charge neutrality condition at the FG and at the sensor surface we have:

At the FG node:
$$\sum_{i=\{FG,liq,S,B,D\}} C_{Surface\ \ to\,i}\,(V_i - V_C) = 0 \qquad \text{(S.1)}$$

At sensor surface: $$\sum_{j=\{Surface,\ MOS,S,B,D\}} C_{FG\ to\ j}\,(V_j - V_{FG}) = 0 \qquad (S.2)$$

Therefore, the FG potential can be derived as a function of all the structural capacitances and voltages as below:

$$V_{FG} = \frac{C_{T-C}}{C_{T-C}\,C_{T-FG} - C_{SG}^2} \times \left[\left(\sum_{i=\{FG,Liq,S,B,D\}} C_{Surface\ to\ i}\,V_i\right) \times \frac{C_{SG}}{C_{T-C}} + \sum_{j=\{Surface,MOS,S,B,D\}} C_{FG\ to\ j}\,V_j\right] \qquad (S.3)$$

With

$$C_{T-C} = C_{CG} + C_{liq} + C_{CS} + C_{CB} + C_{CD} \qquad (S.4)$$

$$C_{T-FG} = C_{SG} + C_{FS} + C_{FB} + C_{FD} \qquad (S.5)$$

Where CT-C and CT-FG are the total capacitance seen from the sensor surface and FG node, respectively. Since all the above capacitance values are nonlinearly change with respect to the physical dimensions, it is difficult to develop a mathematical model which considers the effect of all these dimensions. Instead, we used COMSOL simulation for this purpose. The FG potential as a function of the SG to FG area ratio has been simulated for two different scenarios: when the solution bulk pH varies the surface potential (Fig. S.2a) and when the drain bias changes the effect of the parasitic elements inside the sensor structure (Fig. S.2b). The results show that when the SG/FG area ratio is large, the sensitivity of FG potential to the bulk pH is elevated while the impact of the parasitic elements on the electrical measurement is reduced.

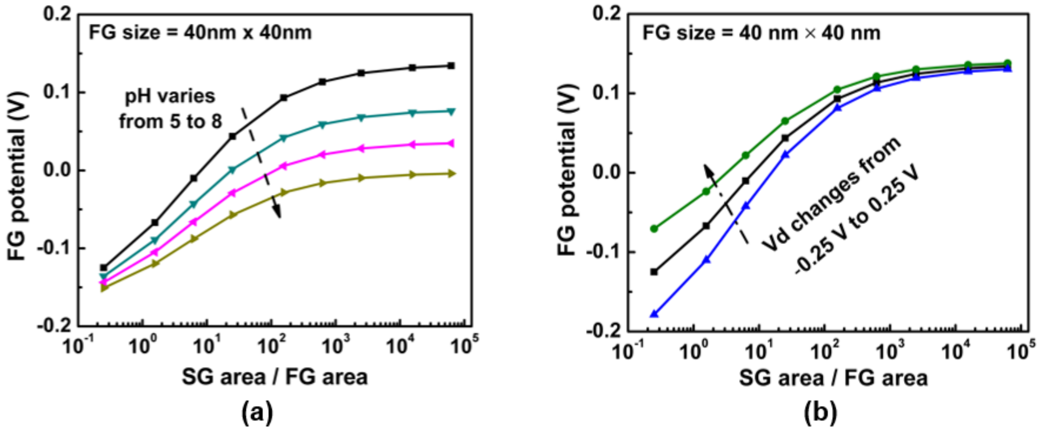

Supplementary Figure S.2: the FG potential as a function of the SG to FG area ratios. Larger the SG/FG area ratios (a) results in to higher sensitivity of FG potential to the change of solution bulk pH, (b) lower the effect of structural parasitic elements on FG potential.

II. Sensitivity equation derivation:

The step by step sensitivity derivation is given in this part:

Equation. 13 in main text can be written as:

$$pH_S = pH_B + \log\left(e^{q\psi_0/kT}\right) - \log\left(1 + 4a^3 c_B \sinh^2\left(\frac{q\psi_0}{2kT}\right)\right) \quad \text{(S.6)}$$

Taking the derivation with respect to pH_B:

$$\frac{dpH_S}{dpH_B} = 1 + \frac{q}{2.3kT}\frac{d(\psi_0)}{dpH_B} - \frac{d\log\left(1 + 4a^3 c_B \sinh^2\left(\frac{q\psi_0}{2kT}\right)\right)}{dpH_B} \quad \text{(S.7)}$$

$$\frac{dpH_S}{dpH_B} = 1 + \frac{q}{2.3kT}\frac{d\psi_0}{dpH_B} - \frac{1}{2.3\left(1 + 4a^3 c_B \sinh^2\left(\frac{q\psi_0}{2kT}\right)\right)}\frac{d\left(1 + 4a^3 c_B \sinh^2\left(\frac{q\psi_0}{2kT}\right)\right)}{dpH_B} \quad \text{(S.8)}$$

$$\frac{dpH_S}{dpH_B} = 1 + \frac{q}{2.3kT}\frac{d\psi_0}{dpH_B} - \frac{4qa^3 c_B \sinh\left(\frac{q\psi_0}{2kT}\right)\cosh\left(\frac{q\psi_0}{2kT}\right)}{2.3kT\left(1 + 4a^3 c_B \sinh^2\left(\frac{q\psi_0}{2kT}\right)\right)}\frac{d\psi_0}{dpH_B} \quad \text{(S.9)}$$

$$\frac{dpH_S}{dpH_B} = 1 + \frac{d\psi_0}{dpH_B}\left(\frac{q}{2.3kT} - \frac{q}{2.3kT}\frac{2a^3 c_B \sinh(\frac{q\psi_0}{kT})}{\left(1 + 4a^3 c_B \sinh^2(\frac{q\psi_0}{2kT})\right)}\right) \tag{S.10}$$

Using eq. 4 we can write:

$$\frac{dpH_S}{dpH_B} = -\frac{C_d}{q\beta_{int}}\frac{d\psi_0}{dpH_B} \tag{S.11}$$

Combining eq. S.10 and eq. S.11:

$$-\frac{C_d}{q\beta_{int}}\frac{d\psi_0}{dpH_B} = 1 + \frac{d\psi_0}{dpH_B}\left(\frac{q}{2.3kT} - \frac{q}{2.3kT}\frac{2a^3 c_B \sinh(\frac{q\psi_0}{kT})}{\left(1 + 4a^3 c_B \sinh^2(\frac{q\psi_0}{2kT})\right)}\right) \tag{S.12}$$

$$\left(-\frac{C_d}{q\beta_{int}} - \left(\frac{q}{2.3kT} - \frac{q}{2.3kT}\frac{2a^3 c_B \sinh(\frac{q\psi_0}{kT})}{\left(1 + 4a^3 c_B \sinh^2(\frac{q\psi_0}{2kT})\right)}\right)\right)\frac{d\psi_0}{dpH_B} = 1 \tag{S.13}$$

$$\frac{d\psi_0}{dpH_B} = \frac{1}{\left(-\frac{C_d}{q\beta_{int}} - \left(\frac{q}{2.3kT} - \frac{q}{2.3kT}\frac{2a^3 c_B \sinh(\frac{q\psi_0}{kT})}{\left(1 + 4a^3 c_B \sinh^2(\frac{q\psi_0}{2kT})\right)}\right)\right)} \tag{S.14}$$

$$\frac{d\psi_0}{dpH_B} = \frac{1}{\left(-\frac{q}{2.3kT} - \frac{C_d}{q\beta_{int}} + \frac{q}{2.3kT}\frac{2a^3 c_B \sinh(\frac{q\psi_0}{kT})}{\left(1 + 4a^3 c_B \sinh^2(\frac{q\psi_0}{2kT})\right)}\right)} \tag{S.15}$$

$$\frac{d\psi_0}{dpH_B} = -2.3\frac{kT}{q}\frac{1}{\left(1+\frac{2.3kTC_d}{q^2\beta_{int}} - \frac{2a^3c_B\sinh(\frac{q\psi_0}{kT})}{\left(1+4a^3c_B\sinh^2(\frac{q\psi_0}{2kT})\right)}\right)} \quad \text{(S.16)}$$

$$\frac{d\psi_0}{dpH_B} = -2.3\frac{kT}{q}\left(\frac{1}{1+\alpha-\delta}\right) \quad \text{(S.17)}$$

With $\alpha = \frac{2.3\,kT\,C_d}{q^2\,\beta_{int}}$ (S.18)

and $\delta = \frac{2a^3c_B\sinh(\frac{q\psi_0}{kT})}{1+4a^3c_B\sinh^2(\frac{q\psi_0}{2kT})}$ (S.19)

β_{int} in eq. S.18 is calculated by site binding model [15]:

$$\beta_{int} = 2.3\,N_S\,a_{H_s^+}\frac{K_b a_{H_s^+}^2 + 4K_aK_ba_{H_s^+} + K_aK_b{}^2}{(K_aK_b + K_ba_{H_s^+} + a_{H_s^+}^2)^2} \quad \text{(S.20)}$$

And C_{diff} is calculated as [25]:

$$C_d = \frac{\varepsilon}{\lambda_D}\frac{\left|\sinh(\frac{zq\psi_0}{kT})\right|}{\left(1+4a^3\,c_B\,\sinh^2(\frac{zq\psi_0}{2kT})\right)\sqrt{\frac{1}{a^3c_B}\ln\left(1+4a^3\,c_B\,\sinh^2(\frac{zq\psi_0}{2kT})\right)}} \quad \text{(S.21)}$$

With $\lambda_D = \sqrt{\frac{\varepsilon kT}{2z^2q^2c_B}}$ (S.22)

III. Simulation result of sensitivity parameters:

The simulated values of the sensitivity parameters of α and δ for various ion sizes are given in Fig. S.3a and S.3b respectively. Fig. S.3c shows the calculated sensitivity for various ion sizes by substituting the simulated α and δ values into our proposed model (eq. 14). Figure S.3d illustrates the sensitivity of surface potential to the bulk pH value obtained directly by simulation which agrees well with the calculated sensitivity which proves the accuracy of our model. The slight change in the behavior of the calculated model can be due to a simplification in our model that ignored the effect of other ions and their sizes. In Fig. S.3c and Fig. S.3d, we have limited the data to the practically valid values of pH sensitivity as discussed earlier.

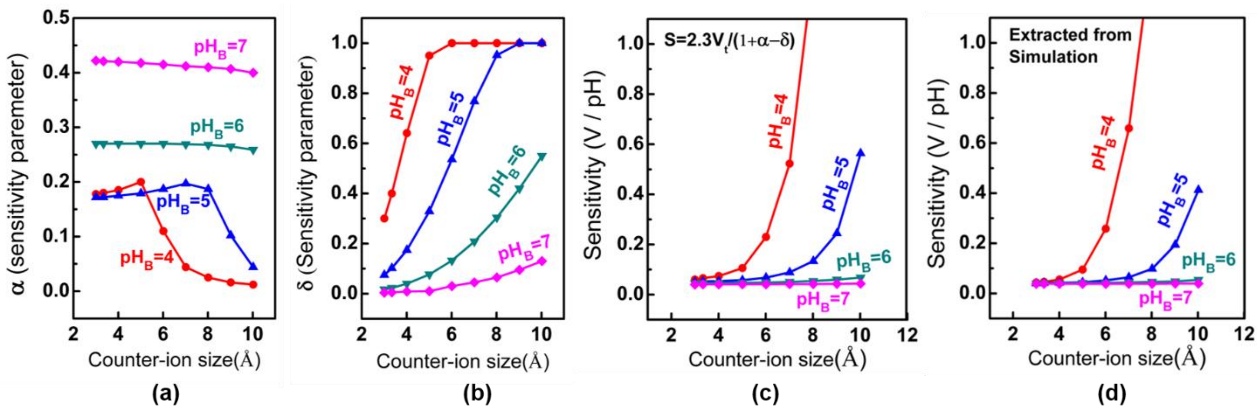

Supplementary Figure S.3: simulated values of parameter α (a), δ (b) and extracted sensitivity using our model (c) for various ion sizes agrees well with the simulated sensitivity directly obtained by simulation (d).

IV. Effect of bulk solution components:

In Fig. S.4, we use two sets of simulation results to illustrate how the counter-ion size affects the potential at the sensor surface for two different salt configurations in bulk solution. Fig. S.4a shows the situation when the solution is buffered with a weak acid and its conjugate base salt with the same concentration while Fig. S.4b illustrates the case when the solution

contains the same concentration of NaCl in addition to the weak acid and its conjugate salt. For the smaller sizes of counter-ions, the surface potential is independent of the size but as the ion size is beyond a specific value (the steric size), the surface potential increases. The steric size is smaller for the pH values farther from pH_{pzc} due to the higher surface charge which attracts more counter-ions near to the surface. As discussed earlier in reference to equation (14), our model loses its validity at very large surface potential. In Fig. S.4a, we only show the values which are practically valid for the surface potential. In Fig. S.4b, the saturation of surface potential is due to the screening effect of Cl^- ions near the surface. When steric effect becomes prominent due to the A^- ions, the surface potential tends to increases due to the buildup charge (H^+) at the oxide surface. At the same time, this increase in surface potential pulls larger amount of Cl^- ions (that has a smaller size) to the surface than the A^- ions. These Cl^- ions screen the effect of H^+ ions at the oxide surface and therefore saturate the surface potential. Diminishing the available sites at the oxide surface as a result of an increase in buildup charge at pH values farther from pH_{pzc} is also another source of saturation of the surface potential.

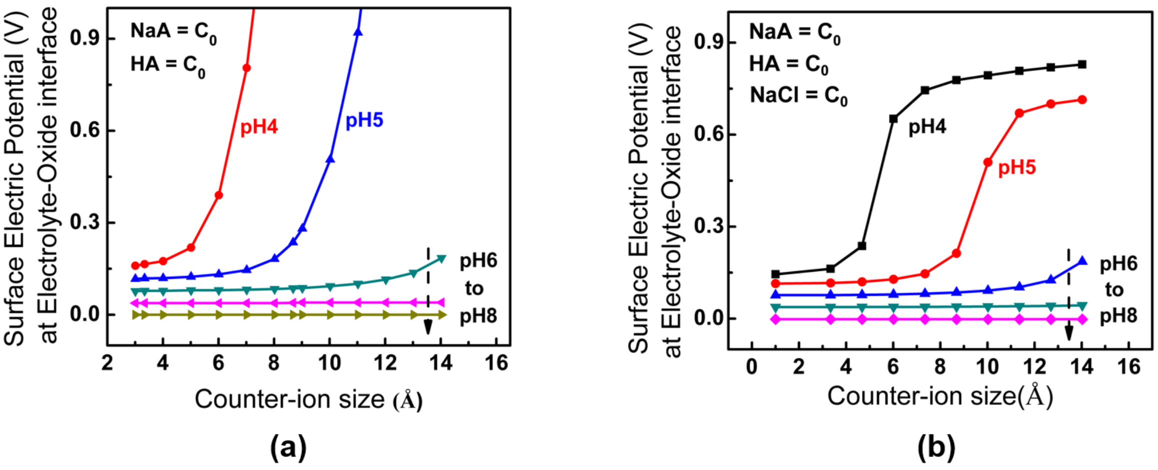

Supplementary Figure 4: The simulated surface potential for various ion sizes at different bulk pH values, (a) the solution is buffered with only weak acid and its conjugate salt, (b) there is an additional salt, NaCl with the same concentration in buffer.

V. Effect of bulk pH:

Figure S.5, demonstrates how the surface charge density is influenced by both bulk pH (pH_B) and counter-ion size. The effect of counter-ion size is more prominent at pH_B farther from pH_{pzc}. Based on the site binding model [16], the surface charge density (σ_0) is zero when $pH_S = pH_{pzc}$ and it reaches its maximum as pH_S decreases (Fig. S.5b).

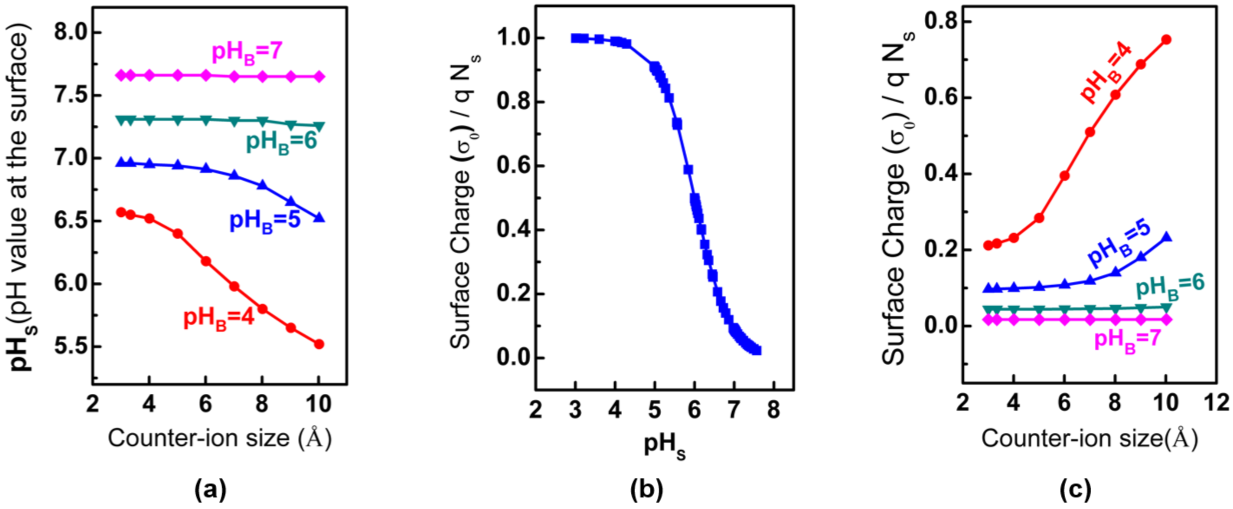

Supplementary Figure 5: (a) The surface pH value (pH_S) is a function of both bulk pH value (pH_B) and ion size, (b) the amount of surface charge (σ_0) varies by pH_S, therefore (c) pH_B and ion size can change the σ_0.

VI. Measurement results of the sensor with different sensing dielectrics:

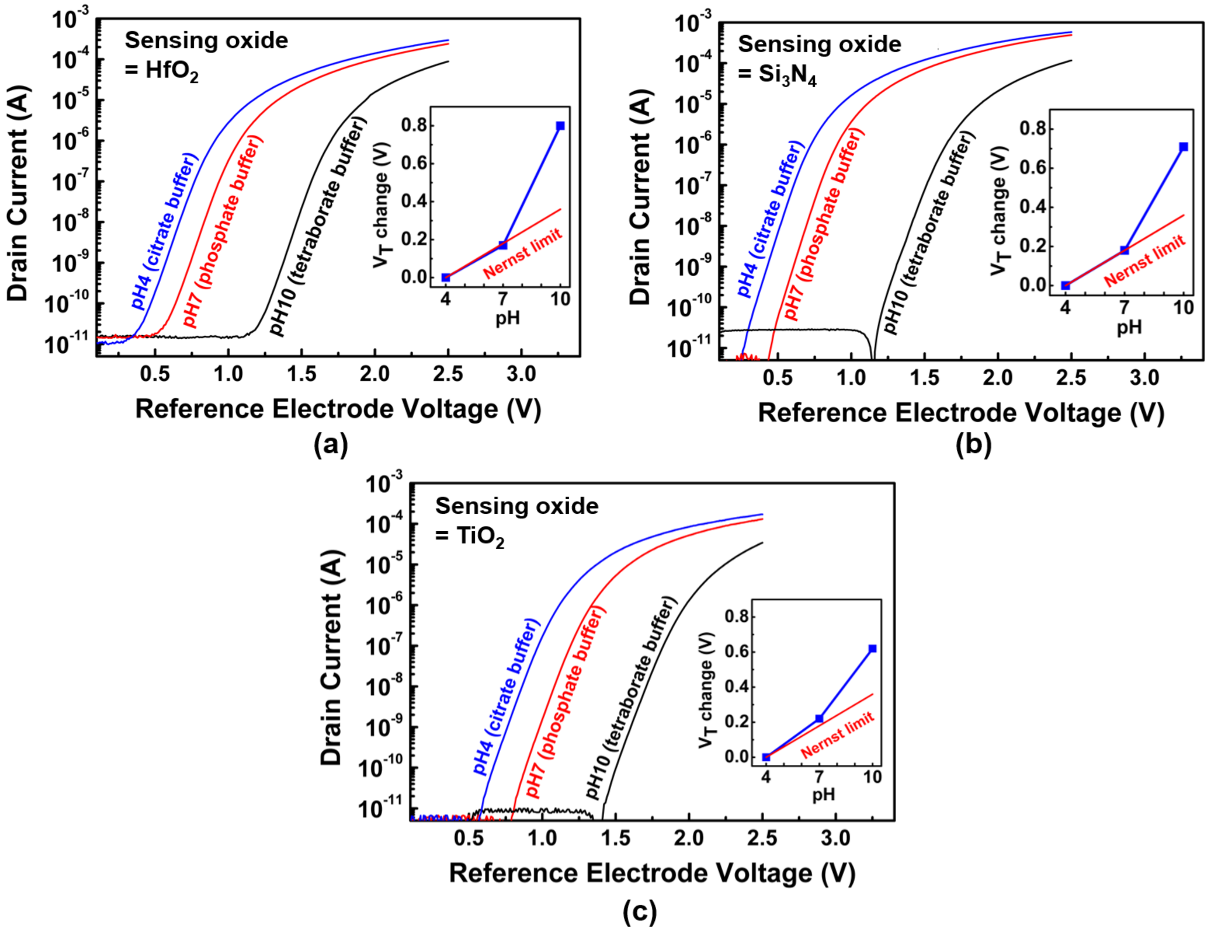

Supplementary Figure 6: Measurement results of our sensor with different dielectrics (a) HfO_2, (b) Si_3N_4 and (c) TiO_2 as the sensing gate oxide.

VII. Additional experimental results:

Figure S.7 shows the capacitance measurement of the sensor when the voltage biases are applied between the reference and silicon bulk electrodes. The reference electrode bias was swept from low voltages (-1V) to high (+2V) and then from high voltages (+2V) to low (-1V). The measured frequency of 400Hz was used for this experiment. The graphs show that there the

hysteresis width in our measurement is very small and does not affect the sensitivity response of our measurement.

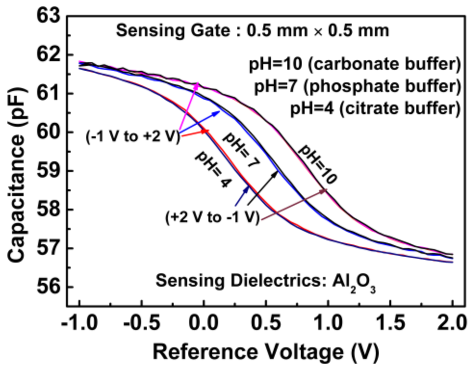

Supplementary Figure 7: Capacitance-Voltage measurement of the sensor between the reference and silicon bulk electrodes. The reference bias varies from negative to positive and then from positive to negative biases.

Figure S.8 illustrates the measurement results of the sensor when TiO2 is used as the sensing dielectric. We can see the response is quiet similar with what we captured when the Al2O3 sensing dielectric is used (Fig. S.9). It also shows the stability of the signal over the time. As it is seen in Fig. S.8b, the signal can track well its previous states while the pH solution changes from 6 to 4, 4 to 10 and then from 10 to 7. This measurement has been done continuously for about 75 minutes. The signal shows small drift over this time, but it is still very low to affect the sensor measurement results.

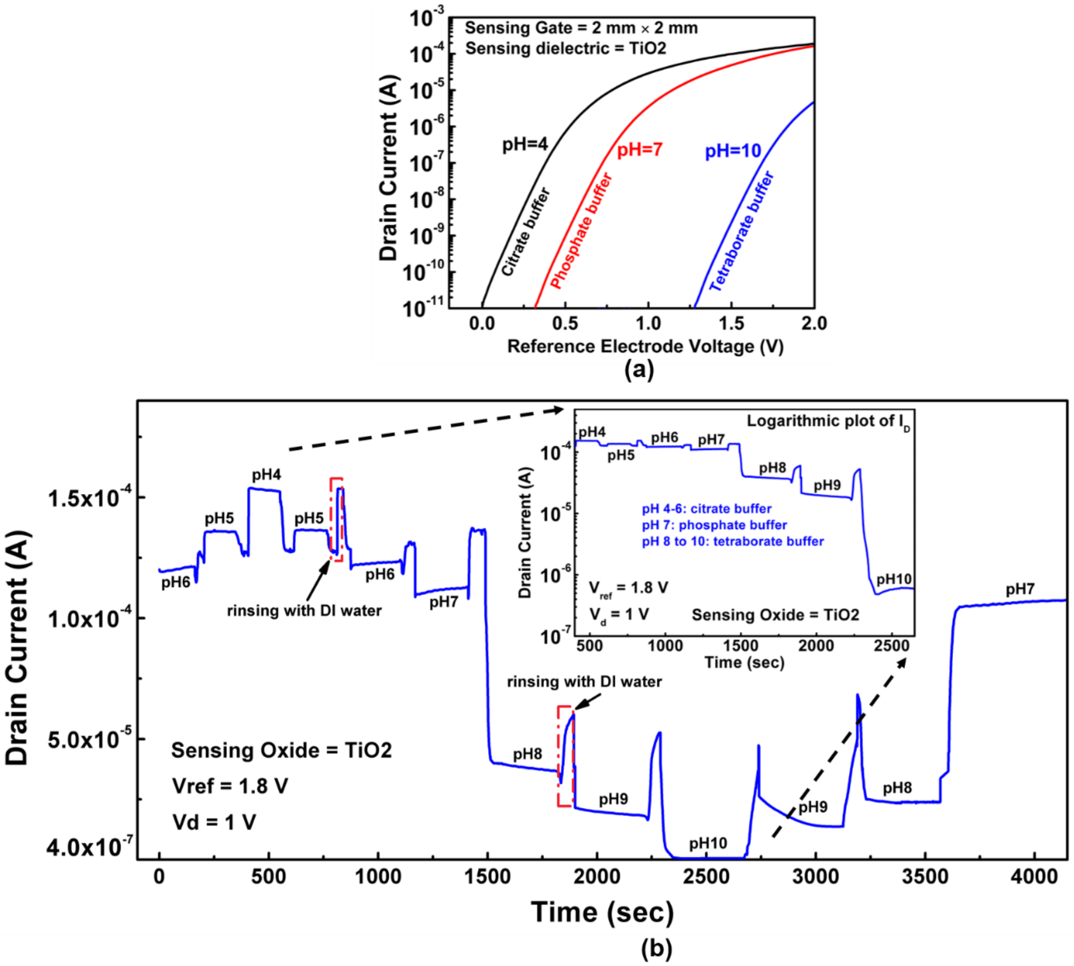

Supplementary Figure 8: The measurement results of the sensor with TiO2 sensing dielectric. (a) the drain current versus the reference electrode bias and (b) the transient response of drain current over the time.

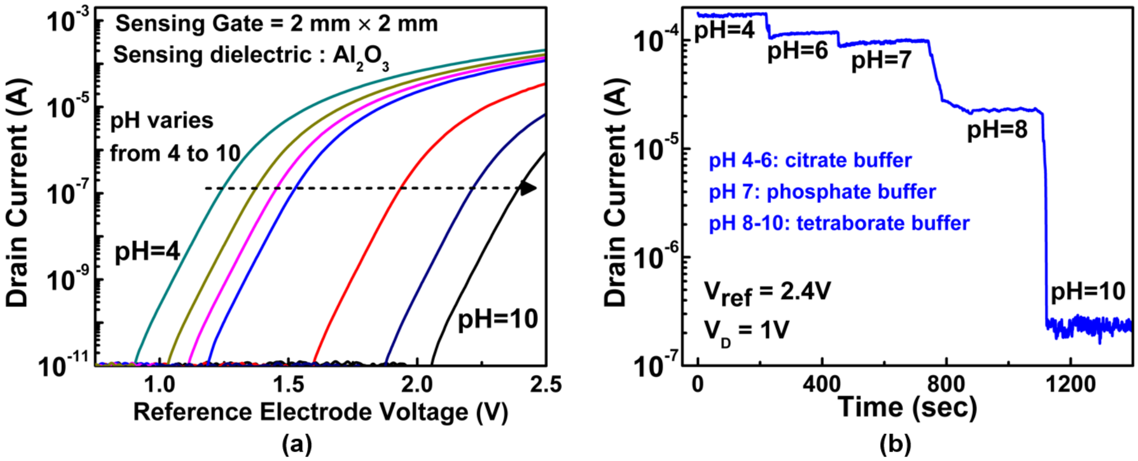

Supplementary Figure 9: The measurement results of the sensor with Al2O3 sensing dielectric when SG/FG = 4×10^4 (SG area = 2 mm × 2 mm, FG area = 5 μm × 20 μm) (a) the

drain current versus the reference electrode bias and (b) the transient response of drain current over the time.

VIII. Buffer solution ingredients:

The ingredients of the buffer solution used in main text figure 6 are listed in table S.1.

Supplementary Table S.1

pH 4	Initial ingredients	concentration
	citric acid mono hydrate (C6H8O7*H2O)	11.76gr = 0.056 M
	sodium hydroxide	2.72 gr= 0.068 M
	sodium chloride	2.57gr=0.044 mole
Final components	Acid Citric = 0.034 M<br>Sodium Citrate = 0.023 M<br>NaCl = 0.044 M	
pH 5	Initial ingredients	concentration
	citric acid mono hydrate (C6H8O7*H2O)	20.26 gr/L = 0.096 M
	sodium hydroxide	7.85 gr/L = 0.196 M
Final components	Acid Citric = 0.031 M<br>Sodium Citrate = 0.065 M	
pH 6	Initial ingredients	concentration
	citric acid mono hydrate (C6H8O7*H2O)	12.54 gr/L = 0.06 M
	sodium hydroxide	6.38 gr/L = 0.029 M
Final components	Acid Citric = 0.051 M<br>Sodium Citrate = 0.009 M	
pH 8	Initial ingredients	concentration
	sodium tetraborate decahydrate (Na2B4O7*10H2O)	4.78gr/L = 0.012 M
	hydrochloric acid	0.74gr/L = 0.02 M
Final components	sodium tetraborate decahydrate = 0.002 M<br>Boric acid = 0.04 M<br>NaCl = 0.02 M	
pH 9	Initial ingredients	concentration
	sodium tetraborate decahydrate, (Na2B4O7*10H2O)	4.78gr/L = 0.012 M
	hydrochloric acid	0.168gr/L = 0.004 M
Final components	sodium tetraborate decahydrate = 0.01 M<br>Boric acid = 0.008 M<br>NaCl = 0.004 M	
pH 10	Initial ingredients	concentration
	sodium tetraborate decahydrate(Na2B4O7*10H2O)	4.78gr/L = 0.012 M
	sodium hydroxide solution	0.74gr/L = 0.018 M
Final components	sodium tetraborate decahydrate = 0.003 M<br>Sodium Borate = 0.036 M	
